# Essential Oil Composition of *Ruta graveolens* L. Fruits and *Hyssopus officinalis* Subsp. *aristatus* (Godr.) Nyman Biomass as a Function of Hydrodistillation Time

**DOI:** 10.3390/molecules24224047

**Published:** 2019-11-08

**Authors:** Ivanka B. Semerdjieva, Marian Burducea, Tess Astatkie, Valtcho D. Zheljazkov, Ivayla Dincheva

**Affiliations:** 1 Department of Botany and Agrometeorology, Agricultural University, Mendeleev 12, 4000 Plovdiv, Bulgaria; v_semerdjieva@abv.bg; 2 Research and Development Station for Aquaculture and Aquatic Ecology, “Alexandru Ioan Cuza” University of Iasi, 700506 Iaşi, Romania; marian.burducea@yahoo.com; 3 Faculty of Agriculture, Dalhousie University, PO Box 550, Truro, NS B2N 5E3, Canada; astatkie@dal.ca; 4 Crop and Soil Science Department, Oregon State University, 3050 SW Campus Way, 109 Crop Science Building, Corvallis, OR 97331, USA; 5 Plant Genetic Research Group, AgroBioInstitute, Agricultural Academy, 8 Dragan Tsankov blvd., 1164 Sofia, Bulgaria; ivadincheva@yahoo.com

**Keywords:** essential oils, 2-nonanone, 2-undecanone, eucalyptol, β-pinene, *cis*-3-pinanone

## Abstract

The aim of this study was to establish the kinetics regression models for yield and composition of *Ruta graveolens* fruit and *Hyssopus officinalis* subsp. *aristatus* aboveground biomass essential oil (EO), collected at different time intervals during the hydrodistillation process. The hypothesis was that collecting the EO fractions during specific time frames may result in EOs with dissimilar composition that may have differential use by the industry. Furthermore, we calculated the kinetics regression models for the composition of EO, isolated by hydrodistillation in a Clevenger-type apparatus and characterized by GC-MS and GC-FID analyses. The EO yield of *R. graveolens* fruits was 0.39% (relative area % of GC-FID chromatogram), with major constituents in the Control fraction (0–90 min) being 2-nonanone, 2-undecanone, and 2-undecanol, representing 65% of the total oil. The highest concentration of 2-nonanone (60%) was found in the 30–60 min oil fraction, the concentration of 2-undecanone (35%) was highest in the Control (0–90 min) fraction, and the concentration of eucalyptol (19%) was highest in the 5–10 min fraction. The EO yield of *H. officinalis* subsp. *aristatus* dried biomass was 1.12%. The major constituents in the Control fraction (0–90 min) of *H. officinalis* biomass were eucalyptol, α-pinene, sabinene, β-pinene, and *cis*-3-pinanone, representing 86% of the total. Eucalyptol (58%) was the highest in the 0–5 min fraction. The highest β-pinene (15%) and *cis*-3-pinanone (20%) contents were found in the 20–40 min fraction. The kinetics regression models that were developed for EO composition of *R. graveolens* were second-order polynominal, Michaelis–Menten, and Exponential decay, while for EO composition of *H. officinalis* subsp. *aristatus* biomass were Exponential decay and Power. The results from this study could benefit the EO industry.

## 1. Introduction

Traditional medicine uses an impressive number of plant-derived natural products [1,2]. *Ruta graveolens* L. (Rutaceae) (common rue) is a tertiary relic, a medicinal plant naturally distributed along the Mediterranean coast, the Balkan Peninsula, and the Crimea [3]. In Bulgaria, the natural populations of *R. graveolens* are low density and have limited distribution. The species is protected by the Bulgarian Biodiversity Act (BBA), and included in the Red Data Book of Bulgaria [4]. The chemical composition of *R. graveolens* is of interest to chemists, pharmacists, and producers of medicinal herb preparations [5,6,7]. Therefore, *R. graveolens* has been either collected from natural habitats or cultivated as a high-value crop in the Mediterranean, North, Central, and South America, China, India, Middle East, and South Africa for a long time [8]. Research has shown that the *R. graveolens* plant contains mainly essential oil (EO) but also coumarins, alkaloids, and flavonoids [5,9,10,11]. *Ruta graveolens* EOs and extracts have broad pharmacological activity. *R. graveolens* products have shown various pharmacological activities such as antimicrobial and cytotoxic activity [6], antibacterial activity [12,13], for treatment of eczema and psoriasis [14]; amenorrhea; activity towards various pathogens; anti-inflammatory, cytotoxic, and antitumor activity [15,16]. Phytochemical studies of *R. graveolens* were most frequently carried out on the entire aboveground biomass (leaves, stems, inflorescences) [12,13,17]. Phytochemical analyses of the composition of the Ruta fruit EO were not found, although reports on the Ruta biomass EO do exist. 2-Undecanone and 2-nonanone were reported as the two major components of the EO of *R. graveolens* aboveground biomass [13,17]. Also, α-pinene, limonene, and eucalyptol were identified as the main monoterpenes in *R. graveolens* biomass EO [18].

*Hyssopus officinalis* L. (Lamiaceae), also known as hyssop, is a perennial plant used as a culinary, medicinal, and essential oil crop, collected in the wild but also cultivated in Asia, Europe, and America [19]. *H. officinalis* EO is widely used in cosmetics, food, and pharmaceutical industries worldwide. Extracts of the species have a wide range of activities, such as antioxidant, antifungal, antimicrobial, and anti-inflammatory activity, they are used for appetite stimulation, and against chronic bronchitis, for treatment of asthma, rheumatic pains, bruises, wounds, anxiety, relaxation of muscles, and so forth [19,20,21,22].

*Hyssopus officinalis* subsp. *aristatus* (Godr.) Nyman (= *H. officinalis* L. subsp. *pilifer* (Pant.) Murb.) is a wild-growing taxon in Western Bulgaria, Znepol region, Vitosha, Predbalkan (Belogradchik) [23]. The range of this subspecies extends from Bulgaria and Northern Greece, northwest to Croatia [24]. Information on the phytochemical composition of *H. officinalis* subsp. *aristatus* and, in particular, the EO content is generally lacking. Džamić et al. [25] identified 30 compounds in the oil of this species. Previous research has shown that the main compounds of EOs were 1,8-cineole, β-pinene, *cis*-3-pinanone, *trans*-pinocamphone, methyl eugenol, and limonene [25,26,27]. There were differences in the composition of hyssop oil depending on various factors such as geographic region, timing of sampling, postharvest handling, and extraction methodology [28,29,30]. The available data on the phytochemical constituents and extractions methods of *H. officinalis* subsp. *aristatus* are insufficient. Overall, the traditional extraction techniques have a number of drawbacks, such as low extraction selectivity, toxic solvent residue in the final extract, and environmental pollution [14,31]. Researchers have been developing environmentally benign extraction methods for EO, such as green extraction techniques [14]. The production of EO fractions over a given time interval has shown to save time and energy and generate EO fractions with unique profile [32].

The purpose of our study was to evaluate the hydrodistillation extraction kinetics of various EO constituents of *R. graveolens* fruits and *H. officinalis* subsp. *aristatus* depending on their elution time. Therefore, we captured and analyzed EO fractions at different timeframes. The underlying hypothesis was that collection of the EO fractions collected at specific time frames will produce EOs with different composition that may have differential use by the EO industry.

## 2. Results

### 2.1. Essential Oil (EO) Content (Yield)

#### 2.1.1. Essential Oil (EO) Content (Yield) of *Ruta graveolens* Fruit in Different Distillation Timeframes (DT) Fractions

The *R. graveolens* dried fruit EO yield of the control treatment (nonstop 90 min distillation) was 0.39% (Table 1). Each fraction had a different yield. Overall, the EO was extracted slowly. Most of the EO came out during the 30–60 min DT timeframe, representing 31% of the total EO yield.

#### 2.1.2. Essential Oil (EO) Content (Yield) of *H. officinalis* subsp. *aristatus* Aboveground Biomass EO Fractions

The EO of *H. officinalis* subsp. *aristatus* aboveground biomass was sequentially collected in seven distinct fractions (Table 1). The control treatment (nonstop 90 min distillation) resulted in 1.12% EO in fresh aboveground biomass. Most of the EO (39% of the total oil yield) was extracted at the beginning of the hydrodistillation (0–5 min) (Table 1).

### 2.2. Essential Oil (EO) Composition of Ruta graveolens Fruits and Hyssopus officinalis subsp. aristatus Biomass

#### 2.2.1. Essential oil (EO) Composition of *R. graveolens* Fruits

Overall, 43 constituents were identified in the six fractions of *R. graveolens* fruit EO (Suppl. Table S1). Ketones (including alkyl ketone) were the most abundant class in the control oil (69.2%) in the 10–30, 30–60, and 60–90 min fractions out of the total EO (Table 2). The major constituents of ketones were 2-nonanone and 2-undecanone (Table 3).

Monoterpenes (acyclic, phenolic, monocyclic, bicyclic) were the most abundant chemical classes in the initial two DT fractions (0–5 min and 5–10 min) (Table 2). Eucalyptol, α-terpineol, and terpinen-4-ol were the constituents with the highest concentration in the 5–10 min DT fraction, and were in much lower concentrations in the fractions obtained in the 10–30, 30–60, and in the 60–90 min DT fractions (Table 4). Also, it was evident that *trans*-pinocarveol and nopinone came out in the first 30 min; these were below the detection limit in later fractions (Table 4).

Sesquiterpenes (bicyclic, tricyclic, monocyclic, and bicyclic sesquiterpenoids) ranged from 19.6% (in the 5–10 min DT fraction) to 3.6% (in the 60–90 min DT fraction). The main EO constituents belonging to the sesquiterpenes are presented in Table 5. β-Caryophyllene, α-caryophyllene, and manoyl oxide were in higher concentrations in the earlier DT fractions compared with the later ones. *δ*-Cadinene, *tau*-cadinol, and *tau*-muurolol had higher concentrations in the 10–30 min DT fraction and they were not found in the 30–60 and 60–90 min DT fractions (Table 5).

Fatty alcohol and fatty acid ester were also found in the *R. graveolens* fruit EO. The concentrations of these were higher in the 0–5 min and in the 10–30 min DT fraction. The 2-undecanol and 2-nonanol were the highest constituents of these fatty alcohols. The highest concentration of undecanol was found in the control EO (Table 3). Fatty acid esters (ethyl decanoate, isopropyl tetradecanoate), phenolic acid ester (α-amyl-cinnamyl acetate), and straight-chain saturated hydrocarbon (*n*-heneicosane, *n*-docosane, *n*-tricosane, *n*-tetracosane, *n*-pentacosane) were found in the EO of the control only. Benzaldehyde, an aromatic aldehyde in *R. graveolens* fruit EO, came out in the highest concentration in the 30–60 min DT.

**Figure 1:**
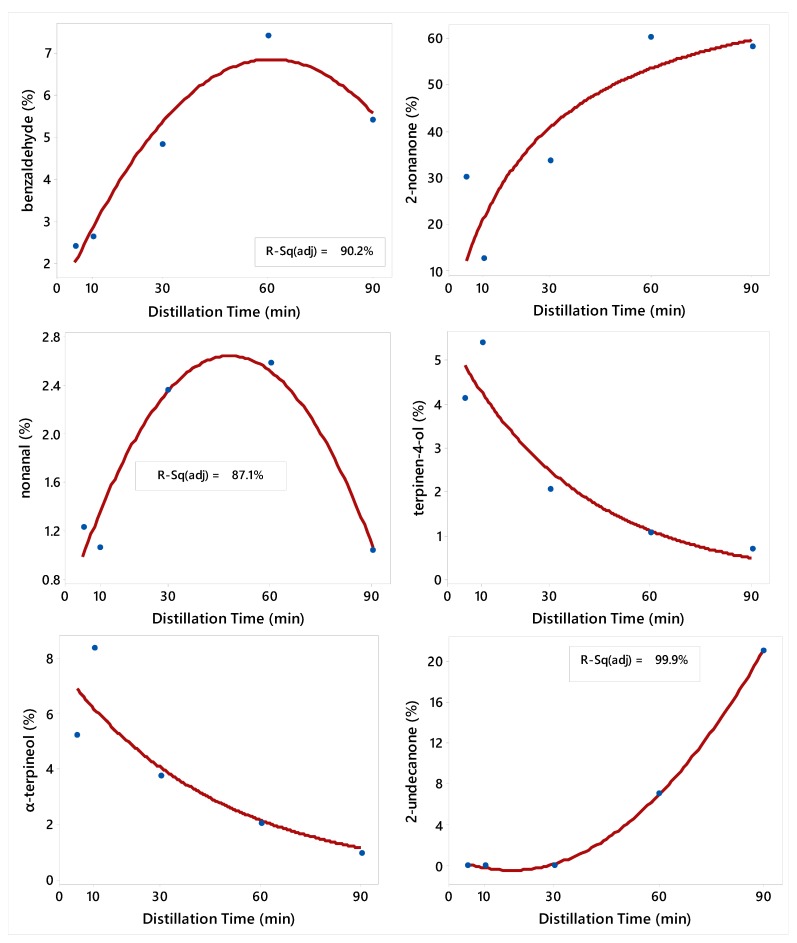
Plot of Distillation Time (DT) vs. the concentrations of six constituents of *R. graveolens* fruit along with the fitted second-order polynomial, Michaelis–Menten, and Exponential regression models. Adjusted R^2^ is given only for the second-order polynomial model, which is linear. The fitted models are: $\hat{Y}=1.12+0.187DT-0.0015DT^{2}$ (benzaldehyde), $\hat{Y}=\frac{77.3DT}{26.8+DT}$ (2-nonanone), $\hat{Y}=0.587+0.085DT-0.0009DT^{2}$ (nonanal), $\hat{Y}=5.56Exp(-0.027DT)$ (terpinen-4-ol), $\hat{Y}=7.67Exp(-0.021DT)$ (α-terpineol), and $\hat{Y}=0.79-0.146DT+0.004DT^{2}$ (2-undecanone).

**Figure 2:**
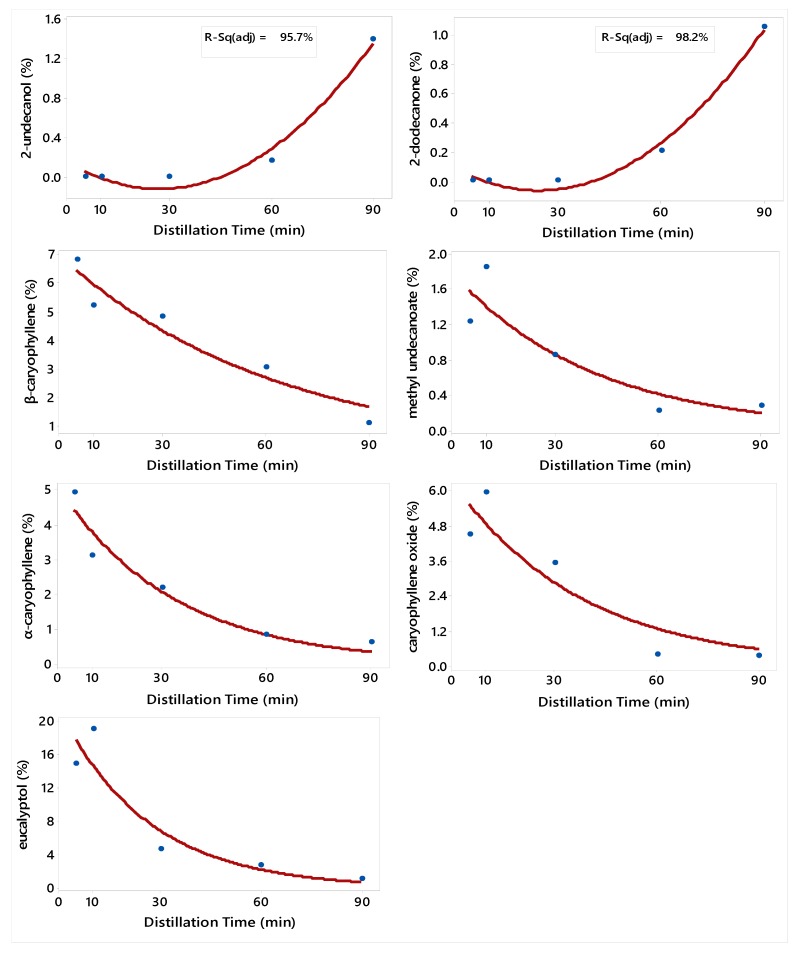
Plot of Distillation Time (DT) vs. the concentrations of six constituents of *R. graveolens* fruit along with the fitted second-order polynomial and Exponential regression models. Adjusted R^2^ is given only for the second-order polynomial model, which is linear. The fitted models are: $\hat{Y}=0.15-0.02DT+0.0004DT^{2}$ (2-undecanol), $\hat{Y}=0.087-0.012DT+0.0003DT^{2}$ (2-dodecanone), $\hat{Y}=6.97Exp(-0.016DT)$ (β-caryophyllene), $\hat{Y}=1.79Exp(-0.025DT)$ (methyl undecanoate), $\hat{Y}=5.11Exp(-0.03DT)$ (α-caryophyllene), $\hat{Y}=6.34Exp(-0.026DT)$ (caryophyllene oxide), and $\hat{Y}=21.4Exp(-0.038DT)$ (eucalyptol).

In order to predict the concentration of EO compounds according to the DT, regression models were developed. The second-order polynomial (Equation (1)) model was the best to describe the relationship between DT and the concentrations of benzaldehyde, nonanal, and 2-undecanone (Figure 1), and 2-undecanol and 2-dodecanone (Figure 2). The Michaelis–Menten (Equation (2)) nonlinear regression model was the best for 2-nonanone (Figure 1); and the Exponential decay (Equation (3)) nonlinear regression model was the best for terpinen-4-ol, and α-terpineol (Figure 1), and β-caryophyllene, methyl undecanoate, α-caryophyllene, caryophyllene oxide, and eucalyptol (Figure 2). There was no regression model that describes the relationship between DT and EO yield and the concentrations of 2-nonanol, *trans*-pinocarveol, nopinone, *δ*-cadinene, *tau*-cadinol, *tau*-muurolol, and manoyl oxide.

#### 2.2.2. Essential Oil (EO) Composition of *H. officinalis* subsp. *aristatus* Aboveground Fresh Biomass

A total of 45 EO constituents were identified in all seven fractions of the *H. officinalis* subsp. *aristatus* oil (Suppl. Table S2). The fractions differed with respect to the concentration of individual constituents. The EO constituents of *H. officinalis* subsp. *aristatus* in this study belonged to several different chemical classes: monoterpenes, sesquiterpenes, and straight-chain saturated hydrocarbons (Table 6).

Class monoterpenes (bicyclic, acyclics, phenolic monoterpenoids, monocyclic) were predominant compounds in the EO of *H. officinalis* subsp. *aristatus*. Monoterpenes came out early in the DT process. Therefore, the concentration of the monoterpenes increased progressively from the 0–5 min to reach the highest concentration in the 60–90 min EO fraction (Table 6), and was the lowest in the control EO. Of the monoterpenes, the highest concentration had the subclass of bicyclic monoterpenes/monoterpenoids (α-pinene, sabinene, β-pinene, eucalyptol, *trans*-pinocarveol, pinocarvone, *cis*-3-pinanone, verbenone, *cis*-verbenol, caryophyllene oxide) (Suppl. Table S2). Of these, eucalyptol had higher concentration in the 0–5 min DT fraction (Table 7). The highest concentrations of *cis*-3-pinanone were observed in the 10–20 and 20–40 min DT fractions.

α-Pinene and sabinene came out also early in the distillation process and decreased gradually in the fractions later in the distillation; their concentrations were the highest in the 0–5 min and in the control oil (Table 7). On the other hand, pinocarvone and *trans*-pinocarveol seem to come out at the same speed throughout the distillation process and had highest concentrations in the control oil (Table 7). β-Pinene elution increased and reached maximum values in the 20–40 min fraction; there was very little elution of this constituent in the 40–60 min timeframe. β-Myrcene eluation was similar to that of β-pinene, but it reached its maximum concentration in the 10–20 min fraction (Table 7). Overall, there were nondetected amounts of α-pinene, sabinene, β-pinene, and β-myrcene in the 60–90 min DT fractions (Table 7).

Monocyclic monoterpenes/monoterpenoids (α-terpinolene, terpinen-4-ol, cryptone, α-terpineol, *trans*-carveol, *cis*-carveol) are the next subclass of the monoterpenes class found in this study. The concentrations of the eucalyptol, α-terpinolene, β-linalool, and *cis*-verbenol were highest in the 0–5 min DT fractions (Table 7 and Table 8), whereas the concentrations of *trans*-pinocarveol and pinocarvone were the highest in the control oil (Table 8). The concentrations of terpinen-4-ol, cryptone, *trans*-carveol, verbenone, and *cis*-carveol reached their max in the 40–60 min DT fraction (Table 9).

Class sesquiterpenes (monocyclic, bicyclic, tricyclic, tricyclic sesquiterpenoid) were the next class of compounds found in *H. officinalis* subsp. *aristatus* EO that reached the highest concentration in the control EO (Table 6). After the first five minutes distillation, the amount of sesquiterpenes decreased in the 60–90 DT fraction. Significant amounts of bourbonene, α-muurolene, bicyclogermacrene, and (-)-spathulenol were eluted in the 0–5 min DT, and were the highest in the control (Table 10). α-Muurolene and bicyclogermacrene were characteristic of EO fractions obtained in the first 40 min (Table 10). These EO ingredients were not identified in the other fractions.

Compounds belonging to the group of straight-chain saturated hydrocarbons (*n*-heneicosane, *n*-docosane, *n*-tricosane, *n*-tetracosane, *n*-pentacosane) were the predominant EO constituents in the control and in the 0-5 min DT EO fraction (Table 6 and Suppl. Table S2).

**Figure 3:**
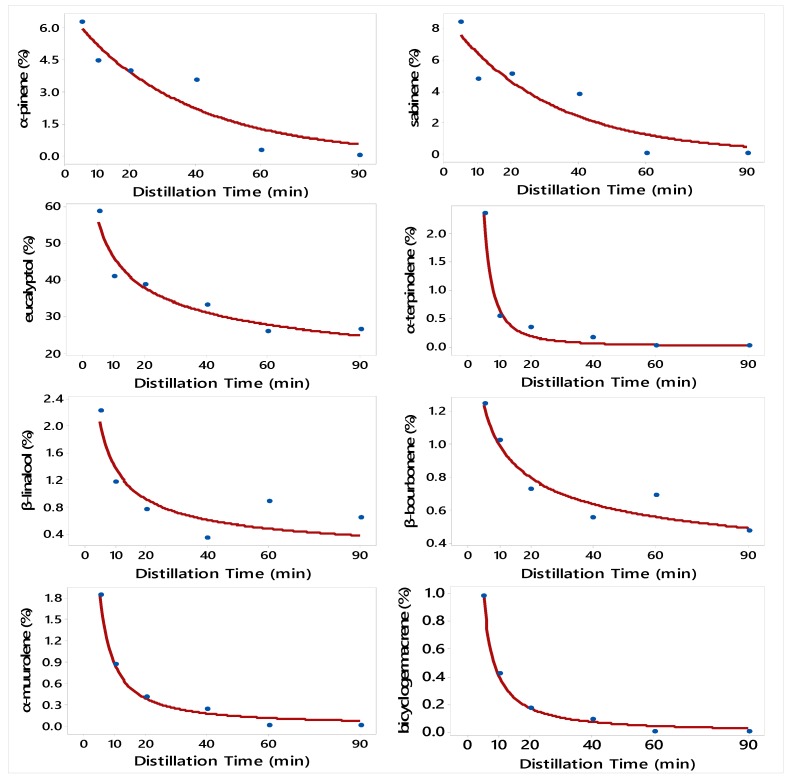
Plot of Distillation Time vs. the concentrations of eight constituents of *H. officinalis* subsp. *aristatus* along with the fitted Exponential and Power nonlinear regression models. The fitted models are: $\hat{Y}=6.88Exp(-0.0284DT)$ (α-pinene), $\hat{Y}=8.95Exp(-0.033DT)$ (sabinene), $\hat{Y}=88.2DT^{-0.2833}$ (eucalyptol), $\hat{Y}=48.4DT^{-1.883}$ (α-terpinolene), $\hat{Y}=5.22DT^{-0.5801}$ (β-linalool), $\hat{Y}=2.05DT^{-0.316}$ (β-bourbonene), $\hat{Y}=11.4DT^{-1.132}$ (α-muurolene), and $\hat{Y}=7.625DT^{-1.266}$ (bicyclogermacrene).

The regression model that describes the relationship between DT and the concentration of each compound of *H. officinalis* is presented in Figure 3. The best regression models were Exponential decay (Equation (3)) for α-pinene and sabinene; and Power (convex) (Equation (4)) for eucalyptol, α-terpinolene, β-linalool, β-bourbonene, α-muurolene, and bicyclogermacrene. There was no regression model that describes the relationship between DT and *H. officinalis* subsp. *aristatus* EO yield and the concentrations of β-pinene, β-myrcene, *trans*-pinocarveol, *cis*-verbenol, pinocarvone, *cis*-3-pinanone, terpinen-4-ol, cryptone, α-terpineol, verbenone, *trans*-carveol, *cis*-carveol, (-)-spathulenol, and caryophyllene oxide.

## 3. Discussion

### 3.1. Essential Oil Content (Yield)

In this study, the EO of *R. graveolens* dried fruits and *H. officinalis* subsp. *aristatus* aboveground fresh biomass were sequentially extracted and characterized by GS-MS. The EO oil of both species was eluted asymmetrically during the distillation process, resulting in EO fractions with different weights. This is the first report on *R. graveolens* and *H. officinalis* subsp. *aristatus* EO fractions eluted and collected in different timeframes subsequent to a grinding of the biomass to considerably accelerate the extraction process. Our results confirmed the hypothesis of this study.

#### 3.1.1. Essential Oil Content (Yield) of *Ruta graveolens* Fruit

In this study, the fraction with the highest yield was obtained at the 30–60 min timeframe (0.12%), while the last fraction (60–90 min timeframe) had the lowest yield (0.03%). As noted in the introduction, phytochemical studies of the two species were most often performed on the whole aboveground plant parts. We did not find studies reporting *R. graveolens* EO from fruits. According to the literature, the EO yield of aerial parts of *R. graveloens* varies between 0.215% [10] and 1.29% [13], depending on the plant organ (leaves, flowers, whole aboveground biomass), type of material (fresh or dried), and environmental factors. For instance, Fredj at al. [33] obtained 0.3% oil yield from fresh leaves and 0.1% from fresh stems; Malik et al. [34] obtained 0.32% from fresh leaves; Orlanda and Nascimento [13] obtained 1.29% from fresh leaves; while the yield of EO from fresh aerial plant parts obtained by Haddouchi et al. [12] was 0.18%. It is rather difficult to compare the oil yield from different plant parts reported in the literature, because EO yield may be a factor of both genetic and environmental factors, but also depends on postharvest handling and the EO extraction method.

#### 3.1.2. Essential Oil Content (Yield) of *Hyssopus officinalis* subsp. *aristatus*

The EO yield of the nonstop distillation control of *H. officinalis* subsp. *aristatus* biomass was 1.12%. A significant amount of the EO (0.44%) was eluted during the first 0–5 min DT, and the lowest (0.05%) during the 40–60 min DT. Therefore, our results suggest that the distillation if *H. officinalis* could be significantly reduced from the reported 3 h DT in the literature [25,27]. Džamić et al. [25] obtained 0.6% EO yield of air-dried plant material of *H. officinalis* subsp. *aristatus* (*w*/*w* dry bases), whereas Piccaglia et al. [27] obtained 0.2%, 0.3%, and 0.4% EO yield from steam-distilled fresh biomass of three different populations of the same species.

The EO yields of *H. officinalis* in this study were much higher than the ones reported in the literature. As mentioned, factors may influence the EO yield of *H. officinalis,* such as harvest time and drying [35], planting densities [36], year of the extraction [37], plant parts [38], and the type of distillation. It is well known that hydrodistillation is more efficient than steam distillation. The results from this study support the concept of reducing energy consumption by controlling the DT.

### 3.2. Essential Oil (EO) Composition of Ruta graveolens Fruit and H. officinalis Subsp. aristatus

#### 3.2.1. Essential Oil (EO) Composition of *Ruta graveolens* Fruit

Forty three (43) constituents were identified in the *R. graveolens* fruit EO. Ketones (including alkyl ketone) was the most abundant class. Aliphatic methyl ketones in *R. graveolens* were reported for the first time more than a century ago by Williams [39]. During the process of distillation, the largest amount of ketones were extracted later in the distillation process, during the 60–90 min DT and also in the control (0–90 min DT). Some of the ketones (2-nonanone, 2-undecanone) were present in all EO fractions, however, the ratio between them varied. The highest concentration of 2-nonanone was found in the 30–60 and the 60–90 DT EO fractions. According to FAO and WHO [40], 2-undecanone is used in the perfumery and flavoring industries. However, it is primarily used as an insect repellent or animal repellent due to its strong odor [40]. 2-Nonanone is a flavoring agent and used in cigarettes, and in other tobacco products [40].

Other ketones (2-tridecanone and 2-decanone) were found in the control EO only. Also, 2-dodecanone, 2-undecanone, and 3-decanone were found in the control EO and in the 60–90 DT fractions (Table 3). The differences in the concentration of ketones contributed to the differences in the EO composition of various fractions, confirming the hypothesis of this study. Ketones play important roles in plants and have commercial uses such as as ingredients in pheromones and natural insecticides in plants [41,42]. They contribute to the aroma of the EO, possess antimicrobial activity [12], and are used as flavoring in cheese and other dairy products [43]. Ketones found in the *R. graveolens* fruit EO in this study were previously reported for *R. graveolens* aboveground biomass as well [10,12,13]. Apparently, all parts of *R. graveolens* contain ketones.

Monoterpenes were the most abundant chemical class in the first and second fractions, with the major constituents of this class being terpinen-4-ol, α-terpineol, eucalyptol, *trans*-pinocarveol, and nopinone (Table 4).

Within the 5–10 min DT fraction, the highest concentration had eucalyptol (19.0%), followed by α-terpineol (8.4%) and terpinen-4-ol (5.4%). Eucalyptol is important and a very popular compound that has been used in a number of ways. Eucalyptol was shown to control airway mucus hypersecretion and asthma via anti-inflammatory cytokine inhibition, it is used in the treatment of nonpurulent rhinosinusitis, it may reduce inflammation and pain when applied topically, and it may enhance skin permeation of bio-affecting agents [44,45].

α-Terpineol plays an important role in some industries. It has a pleasant odor and it is a common ingredient in perfumes, cosmetics, and aromatic scents, and was reported to possess antioxidant, anticancer, and insecticidal properties [46]. Benzaldehyde is also one of the most industrially useful flavor and fragrance agents [47].

#### 3.2.2. Essential Oil (EO) Composition of *Hyssopus officinalis* subsp. *aristatus*

The major constituents of *H. officinalis* subsp. *aristatus* EO in the Control fraction (0–90 min) were eucalyptol, α-pinene, sabinene, β-pinene, and cis-3-pinanone, representing 86% of the total oil.

In a study of *H. officinalis* subsp. *aristatus* in Italy, Piccagli et al. [27] identified three chemotypes: myrtenol (32.6%) and β-pinene (19.3%); β-pinene (24.7%) and eucalyptol (23.1%); and eugenol (43.9%) and limonene (15.9%). The results from our study showed that α-pinene (6.5%), sabinene (9.07%), and *cis*-3-pinanone were also found in the combination of eucalyptol (55.9%) and β-pinene (7.53%). Therefore, our results suggest that the sampled population from Bulgaria belongs to a new chemotype of the species. A similar chemical composition of *H. officinalis* subsp. *aristatus* EO from Serbia was also previously reported [25].

Overall, the EO composition of the control (0–90) and the 0–5 min DT fraction were characterized by the highest specificity, as these EOs contained compounds (e.g., p-cymen-7-ol, perilla alcohol, *tau*-cadinol, *tau*-muurolol, manoyl oxide, *n*-heneicosane, *n*-docosane, *n*-tricosane, *n*-tetracosane, *n*-pentacosane) not found in the other fractions. In this study, the highest concentration of eucalyptol was found in the 0–5 min DT fraction, demonstrating the usefulness of sequential extraction for obtaining EO with different and desirable chemical profiles. As mentioned, eucalyptol has extensive pharmacological activity and it is used against various diseases [44,45,48]. α-Terpinolene and β-linalool have also important commercial uses. They are used as solvents, as a flavoring ingredients in foods, as a fragrance in industrial and household cleaners, soaps, lotions, and perfumes, and as a pesticide in pet sprays, dips, or shampoos [40,49].

The regression model that describes the relationship between DT and the concentration of each compound of *H. officinalis* subsp. *aristatus* is presented in Figure 3. The best regression models were Exponential decay (Equation (3)) for α-pinene, and sabinene; and Power (convex) (Equation (4)) for eucalyptol, α-terpinolene, β-linalool, β-bourbonene, α-muurolene, and bicyclogermacrene. There was no regression model that describes the relationship between DT and *H. officinalis* subsp. *aristatus* EO yield and the concentrations of β-pinene, β-myrcene, *trans*-pinocarveol, *cis*-verbenol, pinocarvone, *cis*-3-pinanone, terpinen-4-ol, cryptone, α-terpineol, verbenone, *trans*-carveol, *cis*-carveol, (-)-spathulenol, and caryophyllene oxide. Similarly, Semerdjieva et al. (2019) used Power (convex) to describe the relationship between distillation time and concentration of α-thujene, α-pinene, α-sabinene, β-pinene, and limonene in *Juniperus* sp. EO.

## 4. Materials and Methods

### 4.1. Plant Material

*Ruta graveolens* L. fruits were obtained from the ex situ collection of the Department of Botany and Agrometeorology at the Agricultural University, Plovdiv, Bulgaria in GPS coordinates 42°08′04.1′’ N 024°46′03.2′’ E., the altitude was 159 m asl. The samples were collected in July 2018 after flowering, when the fruits were ripening. *Hyssopus officinalis* subsp. *aristatus* (Godr.) Nyman was collected from natural population in Western Bulgaria, just before the Buchin Pass in Western Stara Planina (Balkan Mountains). The GPS coordinates of the collection site are: 43°02′14.7” N., 023°07′50.8” E., the altitude was 785 m asl. Samples of *Ruta graveolens* and *Hyssopus officinalis* subsp. *aristatus* biomass used in this study were deposited at the Herbarium of the Agricultural University, Plovdiv, Bulgaria (SOA) [50].

### 4.2. Preparation of Samples for EO Isolation

The collected material of *R. graveolens* was dried at a shady, well-aerated environment. After drying, the *R. graveolens* fruits were separated manually and gently from the biomass samples. The *Ruta* fruits subsamples were generated randomly from each air-dried sample. Fresh *H. officinalis* subsp. *aristatus* samples were placed in a freezer until the essential oil isolation.

### 4.3. Essential Oil (EO) Isolation of the R. graveolens Fruit Samples and H. officinalis subsp. aristatus Biomass Samples

The EO of the aboveground biomass of *H. officinalis* subsp. *aristatus* and *R. graveolens* fruits was extracted via hydrodistillation in two liters distillation units (Laborbio Ltd. Sof, Laborbio.com, Sofia, Bulgaria), at the Research Institute for Roses and Medicinal Plants in Kazanluk, Bulgaria. Each extraction was performed in three replicates. Overall, six samples of R. graveolens and six samples of *H. officinalis* were subjected to hydrodistillation.

Samples of *R. graveolens* consisted of 100 g of air-dried fruits in one liter of water. Samples of *H. officinalis* subsp. *aristatus* consisted of 150 g of fresh aboveground biomass in one liter of water.

The beginning of the distillation was recorded when the first EO drop was deposited in the collecting unit of the apparatus. The EO fractions were captured at different time frames: for *H. officinalis* subsp. *aristatus,* 0–5, 5–10, 10–20, 20–40, 40–60, 60–90 min, and 0–90 min nonstop control. The timeframes for *R. graveolens* were 0–5, 5–10, 10–30, 30–60, 60–90 min, and a nonstop control of 0–90 min. There was no additional EO extracted after 90 min in both species. The eluted EO fractions were collected without interrupting the hydrodistillation process. Therefore, the EO fractions represented the eluted EO constituents within the specific timeframes. First, the oil was measured by volume, transferred into 2 mL vials, and kept in a freezer. Later, the EO was measured on an analytical scale following a separation from the remaining water. The EO samples were stored in a freezer until the time the samples were analyzed. In this study, we report the oil content (yield) based on weight.

### 4.4. Gas Chromatography (GC)-Mass Spectroscopy (MS) Analyses of the EO

The composition of all essential oil samples in two replicates was carried out on a 7890A gas chromatograph (Agilent Technologies Inc., Santa Clara, San Francisco, CA, USA) interfaced with a 5975C mass selective detector (MSD). The individual compounds were separated using a HP-5 ms silica fused capillary column (30 m length × 0.32 mm i.d. × 0.25 µm film thickness). The oven temperature program used was 60 °C for 3 min, increased to 80 °C with 1 °C/min, held for 3 min, then rose with 5 °C/min to 280 °C for 5 min. The flow rate of the carrier gas (He) was maintained at 1.0 mL/min. The injection volume was one µl at split ratios 20:1 (Ruta samples) and 30:1 (Hyssopus samples). The temperatures of the ionization source, the quadrupole, and the injector were 230 °C, 150 °C, and 250 °C, respectively. The MSD was operated in full-scan mode. All mass spectra were acquired in electron impact (EI) mode with 70 eV. The constituents present in the EO samples were identified by comparing their linear retention indices (LRI) and MS fragmentation patterns with those from the National Institute of Standards and Technology (NIST′08) and Adams mass spectra libraries [51]. The estimated LRI were determined using a mixture of a homologous series of aliphatic hydrocarbons from C_8_ to C_36,_ under the same conditions described above.

The GC-FID analysis of the EO was performed with a gas chromatograph 7890A gas chromatograph (Agilent Technologies Inc., Santa Clara, San Francisco, CA, USA) coupled to a flame ionization detector (FID) and HP-5 silica fused capillary column (30 m length × 0.32 mm i.d. × 0.25 µm film thickness). The oven temperature was programmed as mentioned above. The detector and injector temperatures were as follows: 280 °C and 220 °C. The carrier gas was helium at a flow rate of 1 mL/ min. Essential oil samples (1 μL) were injected using the split mode. The percentage composition of compounds (relative quantity) in the studied EO samples was calculated from the GC-FID peak areas using the normalization method, without correction factors.

### 4.5. Statistical Analyses

The effect of distillation time (DT: 5, 10, 30, 60, 90, and Control (nonstop 0–90) min for *R. graveolens* fruit; 5, 10, 20, 40, 60, 90, and Control (nonstop 0–90) min for *H. officinalis*) on essential oil (EO) yield and the concentration of constituents of *R. graveolens* and *H. officinalis* were determined using a one-way analysis of variance. For *R. graveolens*, the constituents were benzaldehyde, 2-nonanone, 2-nonanol, nonanal, *trans*-pinocarveol, nopinone, terpinen-4-ol, α-terpineol, 2-undecanone, 2-undecanol, 2-dodecanone, β-caryophyllene, methyl undecanoate, α-caryophyllene, *δ*-cadinene, caryophyllene oxide, *tau*-cadinol, *tau*-muurolol, manoyl oxide, and eucalyptol. For *H. officinalis* subsp. *aristatus*, the constituents were α-pinene, sabinene, β-pinene, β-myrcene, eucalyptol, α-terpinolene, β-linalool, *trans*-pinocarveol, *cis*-verbenol, pinocarvone, *cis*-3-pinanone, terpinen-4-ol, cryptone, α-terpineol, verbenone, *trans*-carveol, *cis*-carveol, β-bourbonene, α-muurolene, bicyclogermacrene, (-)-spathulenol, and caryophyllene oxide.

For each EO yield and constituent (response variables), the validity of model assumptions was verified by examining the residuals as described in Montgomery [52]. Since the effect of DT was significant (*p*-value < 0.05) on all response variables, multiple means comparison was completed using Tukey’s Multiple Range test at the 5% level of significance, and letter groupings were generated. The analysis was completed using the GLM Procedure of SAS [53].

For *R. graveolens*, the most appropriate regression models that describe the relationships between DT and the concentrations of compounds were second-order polynomial (Equation (1)), Michaelis–Menten (Equation (2)), and Exponential decay (Equation (3)). For *H. officinalis*, the regression models were Exponential decay and Power (convex) (Equation (4)).

(1) $$Y=\beta_{0}+\beta_{1}X+\beta_{2}X^{2}+\varepsilon$$

(2) $$Y=\frac{\theta_{1}X}{\theta_{2}+X}+\varepsilon$$

(3) $$Y=\theta_{1}-\theta_{2}(\exp(-\theta_{3}X))+\varepsilon$$

(4) $$Y=\theta_{1}X^{\theta_{2}}+\varepsilon$$

where *Y* is the dependent (response) variable, *X* is the independent (DT) variable, and the error term ε is assumed to have normal distribution with constant variance.

While the second-order polynomial model (Equation (1)) is linear, the other three models (Michaelis–Menten, Exponential, and Power) are nonlinear, and their parameters were estimated iteratively using the NLIN Procedure of SAS [53], and the fitted models met all adequacy requirements of nonlinear models [54]. The figures as well as the second-order polynomial model fits were completed using Minitab 18 software (Minitab, State College, PA, USA).

## 5. Conclusions

Application of a sequential hydrodistillation method for the extraction of essential oils from *Ruta graveolens* fruit and *Hyssopus officinalis* subsp. *aristatus* generated fractions with different yields and chemical compositions. In *R. graveolens* EO, the yield was different in each fraction, while the highest yield was achieved in the 30–60 min fraction. The highest concentration of the major compounds 2-nonanone and 2-undecanone were found in the 30–60 min oil fraction and in Control, while eucalyptol was highest in the 5–10 min fraction. The fraction with the highest yield of EO of *H. officinalis* subsp. *aristatus* was 0–5 min. Eucalyptol, α-pinene, sabinene, β-pinene, and cis-*3*-pinanone were the major compounds. The highest concentration of eucalyptol was found in the 0–5 min fraction, β-pinene and *cis*-3-pinanone content were highest in the 20–40 min oil fraction. The calculated kinetics regression models (Second-order polynominal, Michaelis–Menten, and Exponential decay, and Power) were able to describe the relationship and predict the concentration of the major compounds in *R. graveolens* and *H. officinalis* subsp. *aristatus*.

## Figures and Tables

**Table 1:** Essential oil yield (%) of *Ruta graveolens* fruits and *Hyssopus officinalis* subsp. *aristatus* biomass.

DT(min)	EO Yield %	DT (min)	EO Yield %
*R. graveolens* Fruits		*H. officinalis* subsp. *aristatus* Biomass	
0–5	0.04 b	0–5	0.44 b
5–10	0.10 b	5–10	0.15 bc
10–30	0.04 b	10–20	0.24 bc
30–60	0.12 b	20–40	0.24 bc
60–90	0.03 b	40–60	0.05 c
Control	0.39 a	60–90	0.20 bc
		Control	1.12a

Within each column, means followed by the same letter are not significantly different at α = 5%. DT: distillation timeframes; EO: essential oil.

**Table 2:** Chemical families of *Ruta graveolens* fruit essential oil (EO) constituents as a function of their extraction during hydrodistillation timeframes (DT).

DT (min)	Control (0–90)	0–5	5–10	10–30	30–60	60–90
Alkyl aldehyde	0.44	2.72	3.67	4.67	3.75	1.07
aromatic aldehyde	0.11	2.44	2.65	4.89	7.27	5.45
Monoterpenes (acyclic, phenolic, monocyclic, bicyclic)	2.79	36.5	47.5	24.1	6.30	3.5
Alkyl ketone; ketone	69.2	30.2	13.5	34.0	67.4	80.0
Alkyl aldehyde	0.14	1.25	1.08	2.40	2.61	1.06
Fatty alcohol	0.92	3.18	3.37	7.49	1.70	2.31
Sesquiterpenes (bicyclic, tricyclic, monocyclic; bicyclic sesquiterpenoid)	8.08	17.8	19.6	19.0	8.09	3.64
Fatty acid ester	7.56	1.25	1.74	0.87	0.22	0.28
Acyclic diterpenoids	3.43	4.39	6.02	3.39	0.42	0.35
Straight-chain saturated hydrocarbon	1.65	nd	nd	nd	nd	nd

nd (no detected).

**Table 3:** The mean concentrations (%) of *Ruta graveolens* fruit EO (benzaldehyde, 2-nonanone, 2-nonanol, nonanal, 2-undecanone, 2-undecanol, 2-dodecanone, and methyl undecanoate) obtained from the six distillation times (DT). Control = nonstop 0–90 min.

DT (min)	Benzaldehyde	2-Nonanone	2-Nonanol	Nonanal	2-Undecanone	2-Undecanol	2-Dodecanone	Methyl Undecanoate
0–5	2.41 d	29.9 d	2.38 b	1.23 c	0.00 d	0.00 c	0.00 d	1.23 b
5–10	2.62 d	12.6 f	2.07 c	1.07 d	0.00 d	0.00 c	0.00 d	1.85 a
10–30	4.80 c	33.5 c	4.58 a	2.36 b	0.00 d	0.00 c	0.00 d	0.86 c
30–60	7.38 a	60.1 a	1.51 d	2.57 a	6.96 c	0.17 c	0.21 c	0.22 d
60–90	5.38 b	57.9 b	0.90 e	1.05 d	21.1 b	1.39 b	1.05 b	0.27 d
Control	0.11 e	25.1 e	0.67 f	0.14 e	35.0 a	5.19 a	2.34 a	2.12 a

Within each column, means followed by the same letter are not significantly different at α = 5%.

**Table 4:** Mean concentrations (%) of *Ruta graveolens* fruit EO (terpinen-4-ol, α-terpineol, trans-pinocarveol, nopinone, and eucalyptol) obtained from the six distillation times (DT). Control = nonstop 0–90 min.

DT (min)	Terpinen-4-ol	α-Terpineol	*trans*-Pinocarveol	Nopinone	Eucalyptol
0–5	4.12 b	5.22 b	2.04 b	2.69 c	14.95 b
5–10	5.37 a	8.35 a	1.60 c	3.03 b	19.04 a
10–30	2.05 c	3.73 c	3.54 a	3.75 a	4.65 c
30–60	1.06 d	2.02 d	0.00 d	0.00 d	2.67 d
60–90	0.71 e	0.93 e	0.00 d	0.00 d	1.03 e
Control	0.62 e	0.57 f	0.00 d	0.00 d	0.29 e

Within each column, means followed by the same letter are not significantly different at α = 5%.

**Table 5:** Mean concentrations (%) of *Ruta graveolens* fruit EO (α-caryophyllene, *δ*-cadinene, caryophyllene oxide, *tau*-cadinol, *tau*-muurolol, β-caryophyllene, manoyl oxide) obtained from the six distillation times (DT). Control = nonstop 0–90 min.

DT (min)	α-Caryophyllene	*δ*-Cadinene	Caryophyllene Oxide	β-Caryophyllene	*tau*-Cadinol	*tau*-Muurolol	Manoyl Oxide
0–5	4.90 a	1.77 c	4.49 b	6.81 a	0.72 b	0.93 c	1.52 c
5–10	3.11 b	2.73 b	5.95 a	5.20 b	0.96 a	1.28 b	6.48 a
10–30	2.16 c	3.74 a	3.53 c	4.82 c	0.95 a	2.02 a	4.16 b
30–60	0.84 d	1.38 d	0.42 d	3.05 d	0.00 d	0.00 e	1.03 cd
60–90	0.60 d	0.80 e	0.35 d	1.10 e	0.00 d	0.00 e	0.40 d
Control	0.23 e	1.06 de	0.46 d	0.83 e	0.46 c	0.45 d	0.95 cd

Within each column, means followed by the same letter are not significantly different at α = 5%.

**Table 6:** Chemical families of *Hyssopus officinalis* subsp. *aristatus* essential oil (EO) constituents as a function of their release during hydrodistillation time frames.

DT (min)	Control (0–90)	0–5	5–10	10–20	20–40	40–60	60–90
Class Compounds							
**Total Monoterpenes**	84.5	88.2	93.9	95.1	91.9	95.3	96.3
Bicyclic monoterpenes monoterpenoids	69.6	73.9	83.5	86.6	78.6	82.6	84.8
Acyclic monoterpenes	3.70	3.90	3.57	3.25	2.39	0.89	0.65
Phenolic monoterpenoids	0.58	0.37	0.15	0.11	0.92	nd	nd
Monocyclic monoterpenes	10.5	10.0	6.76	5.14	9.95	11.9	10.8
**Total Sesquiterpenes**	12	7.97	3.56	1.91	3.52	2.02	1.6
Monocyclic sesquiterpenes	0.19	0.24	0.10	nd	nd	nd	nd
Tricyclic sesquiterpenes	2.57	1.89	1.48	0.96	0.34	0.70	0.49
Bicyclic sesquiterpenes	6.11	4.64	1.47	0.65	3.18	1.32	1.11
Tricyclic sesquiterpenoids	3.1	1.2	0.51	0.30	nd	nd	nd
Straight-chain saturated hydrocarbons	0.80	0.67	nd	nd	nd	nd	nd

**Table 7:** Mean concentrations (%) of *Hyssopus officinalis* subsp. *aristatus* EO (α-pinene, sabinene, β-pinene, β-myrcene, and eucalyptol) obtained from the seven distillation times (DT). Control = nonstop 0–90 min.

DT (min)	α-Pinene	Sabinene	β-Pinene	β-Myrcene	Eucalyptol
0–5	6.28 a	8.45 a	7.90 d	0.32 e	58.6 a
5–10	4.46 b	4.79 b	10.8 c	1.33 c	40.8 c
10–20	3.99 bc	5.12 b	12.7 b	1.72 a	38.6 d
20–40	3.56 c	3.82 c	15.2 a	1.43 b	32.9 e
40–60	0.27 d	0.00 d	0.44 e	0.00 f	25.8 f
60–90	0.00 d	0.00 d	0.00 e	0.00 f	26.3 f
Control	6.58 a	9.07 a	7.53 d	0.65 d	55.9 b

Within each column, means followed by the same letter are not significantly different at α = 5%.

**Table 8:** Mean concentrations (%) of *Hyssopus officinalis* subsp. *aristatus* EO (α-terpinolene, β-linalool, *trans*-pinocarveol, *cis*-verbenol, pinocarvone, and *cis*-3-pinanone) obtained from the seven distillation times (DT). Control = nonstop 0–90 min.

DT (min)	α-Terpinolene	β-Linalool	*trans*-Pinocarveol	*cis*-Verbenol	Pinocarvone	*cis*-3-Pinanone
0–5	2.35 a	2.21 a	2.85 bc	3.49 a	2.91 b	12.78 d
5–10	0.53 c	1.16 c	1.83 d	2.16 c	2.46 c	16.67 b
10–20	0.32 d	0.76 de	1.49 e	1.52 d	1.80 d	18.81 a
20–40	0.15 e	0.34 f	1.27 e	0.29 e	1.24 e	20.06 a
40–60	0.00 f	0.88 d	3.11 b	3.47 a	2.70 bc	14.09 c
60–90	0.00 f	0.64 e	2.80 c	3.02 b	2.57 c	15.81 b
Control	1.29 b	1.80 b	3.62 a	2.37 c	3.24 a	8.61 e

Within each column, means followed by the same letter are not significantly different at α = 5%.

**Table 9:** Mean concentrations (%) of *Hyssopus officinalis* subsp. *aristatus* EO (terpinen-4-ol, cryptone, α-terpineol, verbenone, *trans*-carveol, and *cis*-carveol) obtained from the seven distillation times (DT). Control = nonstop 0–90 min.

DT (min)	Terpinen-4-ol	Cryptone	α-Terpineol	Verbenone	*trans*-Carveol	*cis*-Carveol
0–5	1.49 c	1.69 b	2.91 bc	0.31 d	0.70 d	0.37 d
5–10	1.29 d	0.98 c	2.54 c	0.22 de	0.63 de	0.24 e
10–20	0.92 e	0.42 d	1.93 d	0.19 e	0.55 e	0.16 f
20–40	1.90 b	1.65 b	5.32 a	0.16 e	0.42 f	0.11 f
40–60	2.13 a	2.24 a	3.35 b	3.05 a	2.41 a	1.63 a
60–90	2.12 a	2.23 a	3.07 b	2.34 b	2.07 b	1.11 b
Control	1.74 b	2.30 a	2.94 bc	0.42 c	0.86 c	0.55 c

Within each column, means followed by the same letter are not significantly different at α = 5%.

**Table 10:** Mean concentrations (%) of *Hyssopus officinalis* subsp. *aristatus* EO (β-bourbonene, α-muurolene, bicyclogermacrene, (-)-spathulenol, and caryophyllene oxide) obtained from the seven distillation times (DT). Control = nonstop 0–90 min.

DT (min)	β-Bourbonene	α-Muurolene	Bicyclogermacrene	(-)-Spathulenol	Caryophyllene Oxide
0–5	1.24 b	1.84 b	0.99 b	0.96 c	0.72 c
5–10	1.02 c	0.86 c	0.43 c	0.52 d	0.68 c
10–20	0.73 d	0.41 d	0.17 d	0.30 d	0.22 d
20–40	0.56 de	0.24 e	0.09 e	3.11 a	1.57 a
40–60	0.69 d	0.00 f	0.00 f	1.31 b	0.00 e
60–90	0.48 e	0.00 f	0.00 f	1.09 bc	0.00 e
Control	1.57 a	2.48 a	2.23 a	2.92 a	1.14 b

Within each column, means followed by the same letter are not significantly different at α = 5%.

## Supplementary Materials

The following are available online.

## Abbreviation

FAO	Food and Agriculture Organization;
WHO	World Health Organization;
NLIN	procedure fits nonlinear regression models and estimates the parameters by nonlinear least squares or weighted nonlinear least squares;
GLM Procedure of SAS	The GLM procedure uses the method of least squares to fit general linear models.
