# Supplementary material for: Essential Oil Composition of Ruta graveolens L. Fruits and Hyssopus officinalis Subsp. aristatus (Godr.) Nyman Biomass as a Function of Hydrodistillation Time
Source: Molecules. 2019 Nov 8;24(22):4047. doi: 10.3390/molecules24224047 (PMC6891472; doi:10.3390/molecules24224047)
Supplement: Supplementary file 1 [file molecules-24-04047-s001.pdf]

Supplemental Table 1

Concentration range, percentage of volatile components in *Ruta graveolens* fruits from Bulgaria.

Peak #	RI	Compound name	min	max
1	846	(2E)-2-Hexenal	0.162	2.532
2	901	n-Heptanal	0.135	1.350
3	952	Benzaldehyde	0.107	7.490
4	991	β-Myrcene	nd	0.168
5	1022	o-Cymene	0.145	0.313
6	1024	D-Limonene	0.166	1.214
7	1026	Eucalyptol	0.283	19.303
8	1087	2-Nonanone	12.206	60.380
9	1096	2-Nonanol	0.656	4.662
10	1100	Nonanal	0.141	2.605
11	1135	trans-Pinocarveol	nd	3.609
12	1140	Nopinone	nd	3.821
13	1143	Geijerene	nd	0.903
14	1162	trans-Pinocamphone	nd	8.182
15	1165	n-Nonanol	nd	2.833
16	1174	Terpinen-4-ol	0.607	5.436
17	1185	3-Decanone	nd	1.305
18	1187	α-Terpineol	0.555	8.454
19	1191	2-Decanone	nd	4.113
20	1294	2-Undecanone	nd	35.493
21	1301	2-Undecanol	nd	5.330
22	1388	2-Dodecanone	nd	2.405
23	1417	β-Caryophyllene	0.809	6.896
24	1394	Ethyl decanoate	nd	3.053
25	1424	Methyl undecanoate	0.218	2.183
26	1456	α-Caryophyllene	0.221	4.963
27	1496	2-Tridecanone	nd	2.034
28	1500	α-Muurolene	0.168	1.721
29	1524	δ-Cadinene	0.793	3.805
30	1549	Elemol	nd	0.890
31	1570	Tridecanol	nd	2.423
32	1582	Caryophyllene oxide	0.343	6.022
33	1619	tau.-Cadinol	nd	1.029
34	1638	tau.-Muurolol	nd	2.057
35	1650	β-Eudesmol	nd	3.305
36	1756	α-amyl-Cinnamyl acetate	nd	1.274

37	1828	Isopropyl tetradecanoate	nd	0.579
38	1992	Manoyl oxide	0.395	6.691
39	2100	n-Heneicosane	nd	0.219
40	2200	n-Docosane	nd	0.296
41	2300	n-Tricosane	nd	0.412
42	2400	n-Tetracosane	nd	0.371
43	2500	n-Pentacosane	nd	0.449

nd – not detected

Supplemental Table 2

Concentration range, percentage of volatile components in *Hyssopus officinalis* subsp. *aristatus* aboveground biomass from Bulgaria.

Peak #	RT	RI	Compound name	min	max
1	10.15	933	α-Pinene	nd	6.804
2	10.63	946	Camphene	nd	0.220
3	11.39	969	Sabinene	nd	9.384
4	11.61	974	β-Pinene	nd	15.875
5	12.02	991	β-Myrcene	nd	1.736
6	13.16	1025	p-Cymene	nd	0.923
7	13.20	1026	Eucalyptol	25.036	58.752
8	13.73	1032	(Z)-β-Ocimene	nd	0.532
9	14.12	1043	(E)-β-Ocimene	nd	0.909
10	14.24	1054	γ-Terpinene	nd	0.617
11	15.11	1087	α-Terpinolene	nd	2.377
12	15.55	1096	β-Linalool	0.331	2.233
13	16.84	1135	trans-Pinocarveol	1.240	3.729
14	16.97	1138	cis-Verbenol	0.287	3.529
15	17.48	1160	Pinocarvone	1.216	3.343
16	17.88	1172	cis-3-Pinanone (cis-Pinocamphone)	8.372	20.288
17	17.97	1174	Terpinen-4-ol	0.910	2.169
18	18.20	1183	Cryptone	0.419	2.374
19	18.47	1187	α-Terpineol	1.909	5.433
20	18.88	1195	Myrtenal	nd	1.402
21	19.17	1204	Verbenone	0.155	3.089
22	19.85	1215	trans-Carveol	0.411	2.443
23	19.98	1224	cis-Carveol	0.110	1.646
24	21.26	1289	p-Cymen-7-ol (Cuminol)	nd	0.330
25	21.36	1295	Perilla alcohol	nd	0.307
26	22.10	1324	Myrtenyl acetate	nd	0.463

27	23.55	1374	α-Copaene	nd	0.714
28	23.77	1385	β-Bourbonene	0.469	1.620
29	23.84	1388	β-Cubebene	nd	0.326
30	24.71	1417	β-Caryophyllene	nd	0.518
31	25.62	1456	α-Caryophyllene	nd	0.240
32	26.66	1499	α-Muurolene	nd	2.518
33	26.70	1501	Bicyclogermacrene	nd	2.332
34	27.03	1513	γ-Cadinene	nd	0.216
35	27.14	1524	δ-Cadinene	nd	0.213
36	28.51	1577	(-)-Spathulenol	0.297	3.178
37	28.69	1582	Caryophyllene oxide	nd	1.601
38	30.05	1619	tau.-Cadinol	nd	0.241
39	30.36	1638	tau.-Muurolol	nd	0.348
40	37.44	1992	Manoyl oxide	nd	0.602
41	39.31	2100	n-Heneicosane	nd	0.151
42	41.13	2200	n-Docosane	nd	0.161
43	42.85	2300	n-Tricosane	nd	0.194
44	44.49	2400	n-Tetracosane	nd	0.145
45	46.08	2500	n-Pentacosane	nd	0.217

nd – not detected
